# Research advances and trends in the surgical treatment of carpal tunnel syndrome from 2003 to 2022: A CiteSpace-based bibliometric analysis

**DOI:** 10.3389/fneur.2023.1124407

**Published:** 2023-04-06

**Authors:** Daqiang Zheng, Zhiming Wu, Lu Li, Sichao Chen, Jianjun Chang

**Affiliations:** ^1^Third Hospital of Shanxi Medical University, Shanxi Bethune Hospital, Shanxi Academy of Medical Sciences, Tongji Shanxi Hospital, Taiyuan, China; ^2^Department of Orthopedics, Shanxi Bethune Hospital, Shanxi Academy of Medical Sciences, Tongji Shanxi Hospital, Third Hospital of Shanxi Medical University, Taiyuan, China

**Keywords:** surgery, carpal tunnel syndrome (CTS), bibliometric analysis, CiteSpace, nerve injuries

## Abstract

**Background:**

Carpal Tunnel Syndrome (CTS) is one of the most common peripheral neuropathies. The typical symptoms are tingling and numbness in the median nerve distribution of the hand. Current treatment for CTS includes general conservative treatment and surgical treatment. Surgical treatment plays a crucial role in the management of CTS, but little bibliometric analysis has been conducted on it. Therefore, this study aimed to map the literature co-citation network using CiteSpace (6.1 R4) software. Research frontiers and trends were identified by retrieving subject headings with significant changing word frequency trends, which can be used to predict future research advances in the surgical treatment of CTS.

**Methods:**

Publications on the surgical treatment of CTS in the Web of Science database were collected between 2003 and 2022. CiteSpace software was applied to visualize and analyze publications, countries, institutions, journals, authors, references, and keywords.

**Results:**

A total of 336 articles were collected, with the USA being the major publishing power in all countries/regions. JOURNAL OF HAND SURGERY AMERICAN VOLUME was the journal with the most published and co-cited articles. Based on keyword and reference co-citation analysis, keywords such as CTS, surgery, release, median nerve, and diagnosis were the focus of the study.

**Conclusion:**

The results of this bibliometric study provide clinical research advances and trends in the surgical treatment of patients with CTS over the past 20 years, which may help researchers to identify hot topics and explore new directions for future research in the field.

## 1. Introduction

Carpal tunnel syndrome (CTS) is one of the most common peripheral neuropathies, and it is generally accepted that the median nerve is damaged by pressure as it passes through the carpal tunnel (1). However, the exact cause of this neuropathy is still unknown. Typical symptoms of CTS include nocturnal pain with tingling and numbness in the distribution of the median nerve in the hand, and further progression without proper treatment may result in atrophy of the interosseous muscles of the hand, with end-stage hand disability and loss of work capacity (2). This can lead to disability and incapacity, seriously affecting the patient’s quality of life.

Non-surgical conservative treatments include acupuncture, electrotherapy and medication and are generally used in the early stages of CTS (3). However, it is worth noting that, on the one hand, there are no studies that point to the existence of the best treatment or the best combination of treatment measures (4), on the other hand, conservative treatments can only delay the progression of the disease in the short term. Studies have shown that the release of the carpal tunnel and the reinnervation of the skin associated with it are significantly associated with improved symptoms and function (5). For this reason, surgical treatment remains an important tool in the treatment of moderate to severe CTS, with its unique advantages (6, 7). The traditional surgical treatment is open transverse carpal ligament release. The traditional surgical treatment is open transverse carpal ligament release, which remains the standard of care for CTS because of its effectiveness (8). However, with the development of new devices, the traditional open treatment is gradually being replaced by minimally invasive techniques with shorter operative times, less trauma, smaller postoperative scars and shorter incision healing times (9, 10).

Bibliometrics refers to the cross-cutting science of quantitative analysis of all knowledge vehicles using mathematical and statistical methods and is a statistical analysis and quantitative tool for research publications (11). In the past, studies have confirmed the safety and effectiveness of surgical treatment of CTS.9 However, little bibliometric analysis of studies related to the surgical treatment of CTS has been reported. Therefore, there is a need to understand the current state of research on the surgical treatment of CTS as a whole. This study aimed to assess research trends in the surgical treatment of CTS and to provide a macro review of the extensive academic literature over the past decade through a bibliometric analysis.

CiteSpace is a visual literature analysis software based on the JAVA language environment created by Professor Chaomei Chen and is now widely used in many fields such as information science, education and medicine (12). It features a co-occurrence network graph of countries, institutions, authors, keywords, cited journals, cited authors and cited references (13, 14). In this study, CiteSpace was used to conduct a bibliometric analysis to explore global advances and research trends in the surgical treatment of CTS over the past 20 years (2003 to 2022), with all eligible literature obtained from the Web of Science database.

## 2. Data and methods

### 2.1. Data collection

All data in this study were obtained from the Web of Science database on December 1, 2022. The literature search measurement strategy was ((TS = (carpal tunnel syndrome)) AND TS = (treat)) AND TS = (surgery), with literature publication, dates from 1 January 2013 to 1 December 2022; in addition, the country, category and language of publication were not restricted. The final search yielded 336 documents: exported as “plain text files” with the record “full record with cited references.”

### 2.2. Inclusion criteria

Inclusion criteria were: (1) Peer-reviewed published original articles on surgical treatment of CTS, including basic and clinical research; (2) reviews on surgical treatment of CTS; and (3) articles published from 2003 to 2022.

### 2.3. Exclusion criteria

Exclusion criteria were: (1) articles collected by hand and telephone; (2) articles not officially published; (3) conference abstracts and corrigendum documents; (4) repeated publications; and (5) unrelated articles.

### 2.4. Methods

The documents were analyzed econometrically using CiteSpace (6.1R4). Node types were selected for country, institution, author, keyword, cited journal, cited author and cited reference, Time Slicing was set to Form 2003 JAN to 2022 DEC, time partitioned to 1 year, number of Years Per Slice (1), all options in Term Source were selected, one node type was selected at a time, Selection Criteria (g-index, *k* = 25), and Pruning (Pathfinder). The visual knowledge graph consists mainly of nodes and links. Each node in the map represents an element, such as an author, keyword, institution, etc. The size of the nodes usually indicates the frequency of occurrence or citation, and the different colors of the nodes indicate different years, with different colored circles running from the inside to the outside of the nodes indicating the years 2003 to 2022. In addition, lines between nodes indicate collaboration, co-occurrence, or co-referencing relationships. The purple circles represent centrality, and nodes with high centrality are often considered to be key points in a particular domain.

## 3. Results and discussion

### 3.1. Global publishing trends

#### 3.1.1. Countries and regions

**Figure 1:**
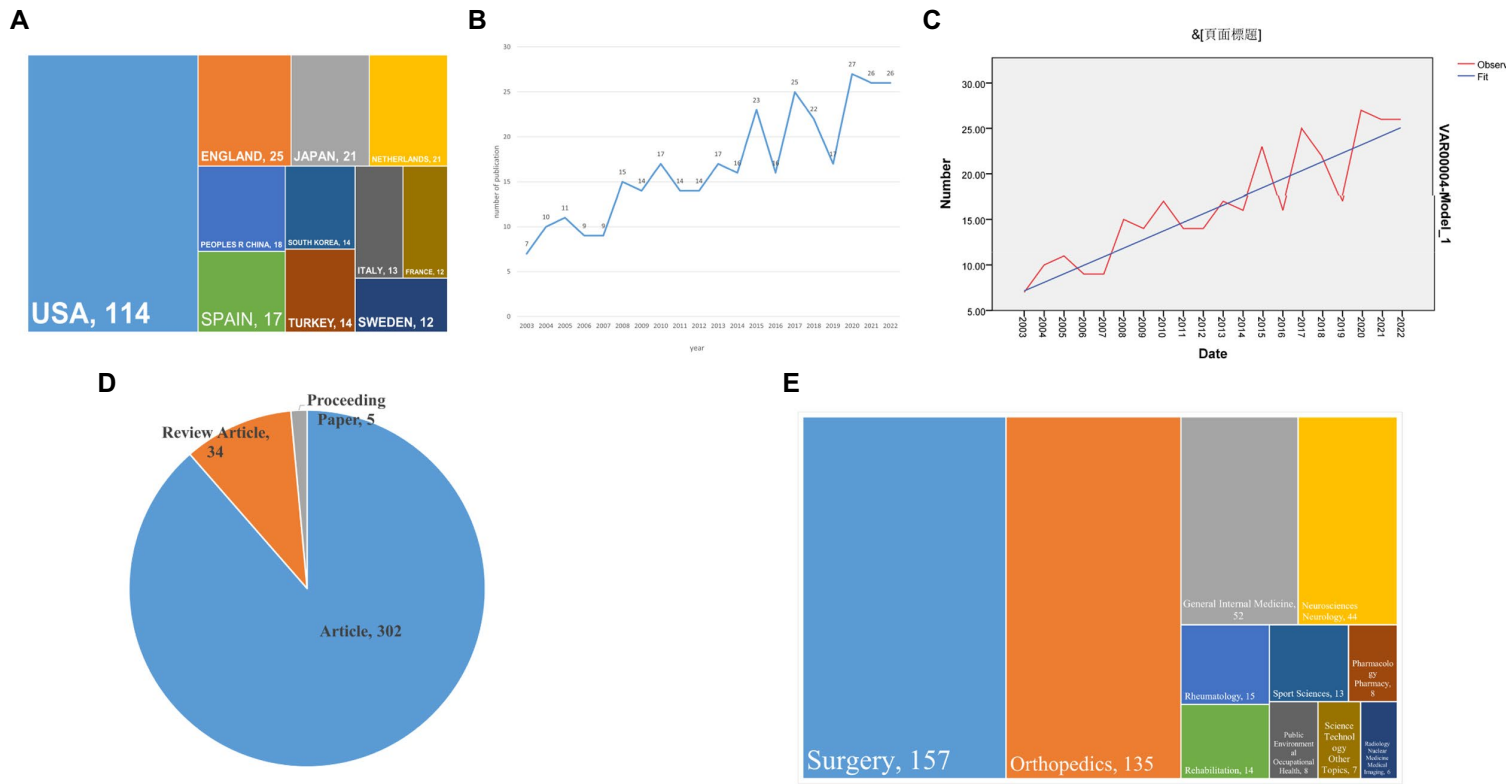
Global characteristics in the surgical treatment of CTS. **(A)** Volume of publications on surgical treatment of CTS by country and region from 2003 to 2022. **(B)** Annual volume of publications on surgical treatment CTS from 2003 to 2022. **(C)** Future publication forecast curve. **(D)** Article-genre breakdown on surgical treatment of carpal tunnel syndrome from 2003 to 2022. **(E)** WoS catalog classification of research areas.

A total of 336 articles that met our search criteria were retrieved from the Web of Science database. The volume of articles published in the field of surgical treatment of CTS research by country and region worldwide was analyzed (Figure 1A). The USA contributed the most articles in this area (114, 34.03%), followed by the UK (25, 7.46%), Japan (21, 6.27%), the Netherlands (21, 6.27%), and China (18, 5.37%). The data was used in CiteSpace software for visual analysis.

#### 3.1.2. Annual publication volume

A search within the Web of Science database by subject term revealed a total of 336 articles included in it, along with a count of the number of articles published in each 1-year period (Figure 1B).

The study found a steady upward trend in the number of publications from 2003 to 2022, with the highest number of publications (27) in 2020 and a plateau in the last 3 years. The trend in publications suggests that the surgical treatment of CTS has been gaining increasing attention from researchers over the last 20 years. It can be predicted that the annual number of publications in this field will continue to trend upwards.

#### 3.1.3. Forecasting global publishing trends

The trend extrapolation trend analysis using SPSS software predicts future publishing trends in the field (Figure 1C). Indicates an upward trend in the number of publications in the future.

#### 3.1.4. Type of literature

Dividing all articles by category (Figure 1D), the highest of these 336 articles was Article (302, 89.88%), followed by Review Article (34, 10.12%), and Proceeding Paper (5, 1.5%).

#### 3.1.5. Areas of research

In the Web of Science database, the study covered 32 fields (Figure 1E), with the majority of the literature concentrated on “Surgery” (157; 46.73%), “Orthopedics” (135; 40.18%), “General Internal Medicine” (52; 15.48%) and “Neurosciences Neurology” (44; 13.10%), (135; 40.18%), “General Internal Medicine” (52; 15.48%) and “Neurosciences Neurology” (44; 13.10%), indicating that the Surgical treatment of CTS is a multifaceted and multidisciplinary field that covers a wide range of societal benefits.

#### 3.1.6. Published journals

All 336 articles were published in 38 journals. The top 10 journals in terms of publication volume are shown in Table 1, among which JCR Q1 4, Q2 1, Q3 3, Q4 2, and the overall publication quality is high.

**Table 1:** The TOP-10 journals with the largest number of publications.

Rank	Journal	Count	IF	JCR (2021)
1	JOURNAL OF HAND SURGERY AMERICAN VOLUME	47	2.342	Q3
2	PLASTIC AND RECONSTRUCTIVE SURGERY	13	5.169	Q1
3	BMC MUSCULOSKELETAL DISORDERS	8	2.562	Q3
4	HAND SURGERY REHABILITATION	7	1.419	Q4
5	JOURNAL OF HAND SURGERY EUROPEAN VOLUME	7	2.206	Q3
6	JOURNAL OF ORTHOPEDIC SPORTS PHYSICAL THERAPY	6	6.276	Q1
7	JOURNAL OF PLASTIC SURGERY AND HAND SURGERY	6	1.295	Q4
8	MUSCLE NERVE	6	3.852	Q2
9	CLINICAL ORTHOPEDICS AND RELATED RESEARCH	5	4.755	Q1
10	COCHRANE DATABASE OF SYSTEMATIC REVIEWS	5	12.008	Q1

### 3.2. Collaborative network analysis

After importing 336 articles into the CiteSpace (6.1 R4) software, we deduplicated and cleaned the data and found zero duplicates, and a total of 336 articles were extracted for analysis. All articles were published in 179 journals by 316 researchers from 157 countries/regions.

#### 3.2.1. Analysis of national/regional collaboration networks

**Figure 2:**
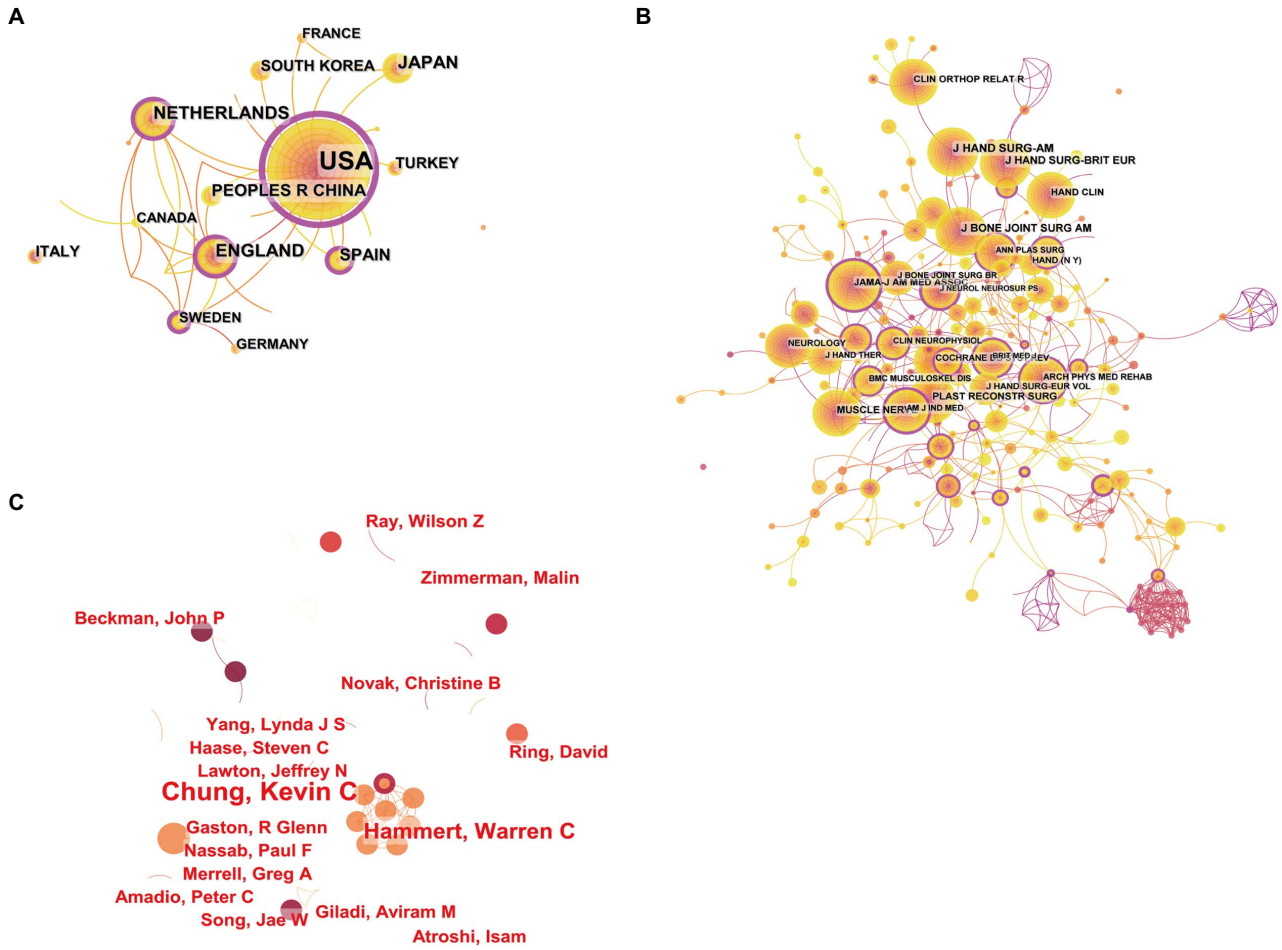
Analysis of collaborative networks on surgical treatment of carpal tunnel syndrome from 2003 to 2022. **(A)** The collaborative network of National/regions. **(B)** The collaborative network of authors. **(C)** The collaborative network of journals.

The national collaboration networks from 2003 to 2022 were visualized through CiteSpace software and mapped (Figure 2A), showing countries that published at least 10 articles. The country with the highest number of publications is the USA with 114 articles, accounting for 33.93% of total publications, and maintains collaborative relationships with 17 other countries, with the top 5 also including the UK (25 articles), Japan (21 articles), the Netherlands (21 articles), and China (19 articles). China is the only developing country among them.

**Figure 3:**
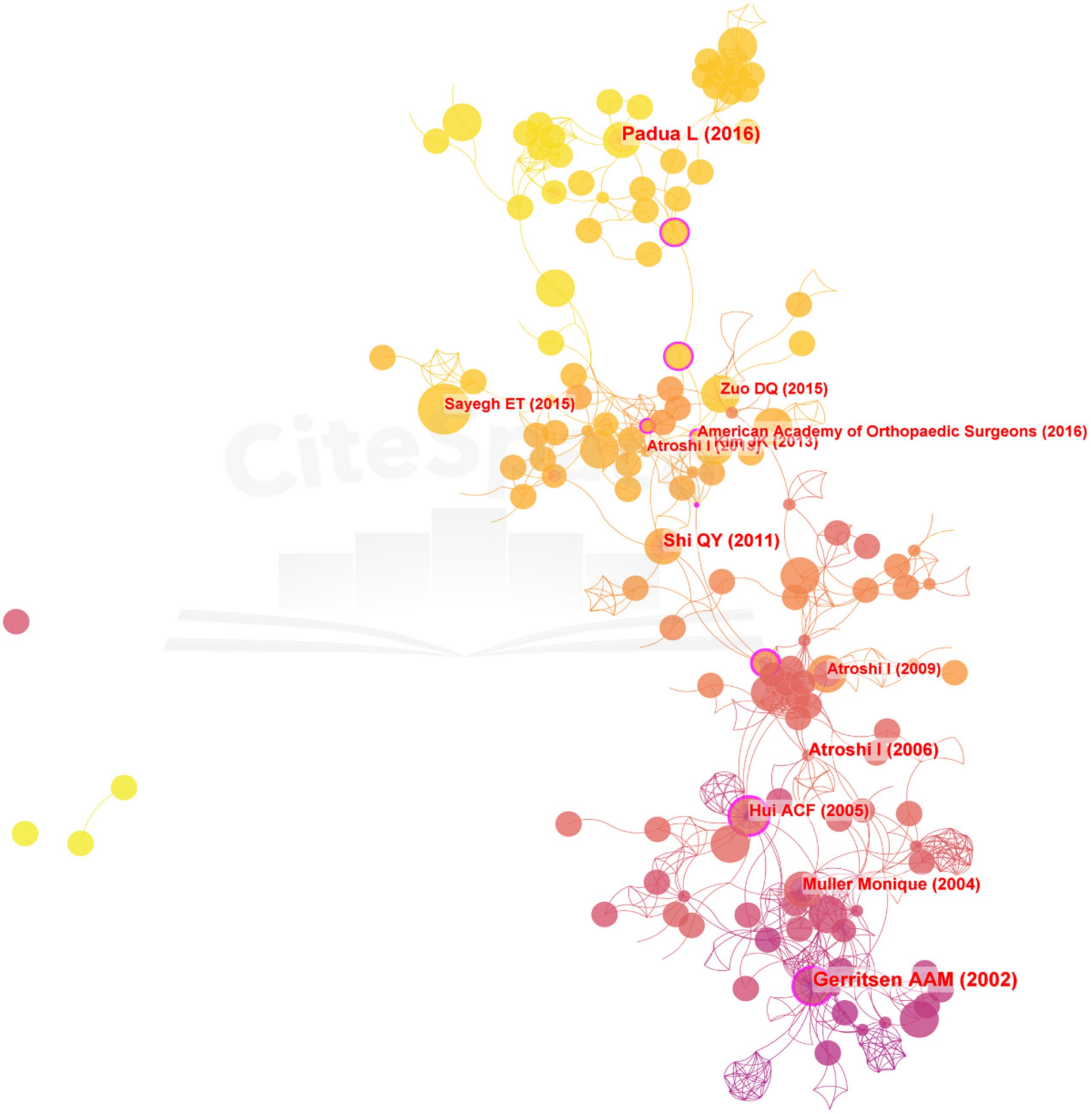
Reference co-occurrence network in the field of surgical treatment of CTS research.

Higher centrality values, also known as mediated centrality, indicate the more active and closer role that nodes play in collaborative relationships with other nodes, and individuals with high centrality are marked with a purple halo in the graph. The USA (0.66) has a high intermediary centrality (Figure 3), indicating that it plays an important bridging role in the network of countries cooperating in this area. Japan (0.00) and China (0.01) do not have high centrality, although they have a higher number of publications.

#### 3.2.2. Author collaboration network analysis

With all 336 articles originating from 316 researchers, we generated an author collaboration network for this research study (Figure 2B). In the author collaboration network, each node represents an author, the line between the nodes represents a collaborative relationship between two individuals, and the color of the line represents the year of collaboration. As shown in the figure, there is less collaboration between researchers and between teams of researchers in this research area. At the same time, some scholars have formed collaborative networks centered on themselves and have a more consistent article output (Figure 2C). Overall, there is a lack of collaboration between many scholars and teams, with some important teams with a large number of early publications even phasing out of the field.

#### 3.2.3. Analysis of journal collaboration networks

The names and number of publications in the last 10 years involving surgical treatment of CTS are shown in Table 1, with JOURNAL OF HAND SURGEGY AMERICAN VOLUME being the most productive journal with 47 articles, followed by PLASTIC AND RECONSTRUCTIVE SURGERY with 13 articles, both of which are specialist journals in the neighborhood of the specialty. We also used CiteSpace to generate a citation co-occurrence graph for journals (Figure 2B). The nodes in the graph represent journals, and the connections between the nodes represent citation relationships. In addition, the purple ring outside the node represents the centrality of the journal, and nodes with high centrality are considered to be key journals. The top ranked journals for frequency and centrality are JOURNAL OF HAND SURGEGY AMERICAN VOLUME and JOURNAL OF NEUROLOGY NEUROSURGERY AND PSYCHIATRY, respectively. Of all the citations in JOURNAL OF HAND SURGERY AMERICAN VOLUME, one of the articles that has received more attention is a questionnaire analysis of members of the American Society for Surgery and Hand Surgery, in which the authors investigate the location of the procedure, the use of preoperative electrophysiological aids to diagnosis, the use of preoperative antibiotics, the choice of surgical incision, the choice of anesthesia, and the management of the patient postoperatively. The questionnaire survey is, in our opinion, of interest to researchers worldwide studying the surgical treatment of CTS.

### 3.3. Keyword co-word analysis

#### 3.3.1. High frequency keywords

**Figure 4:**
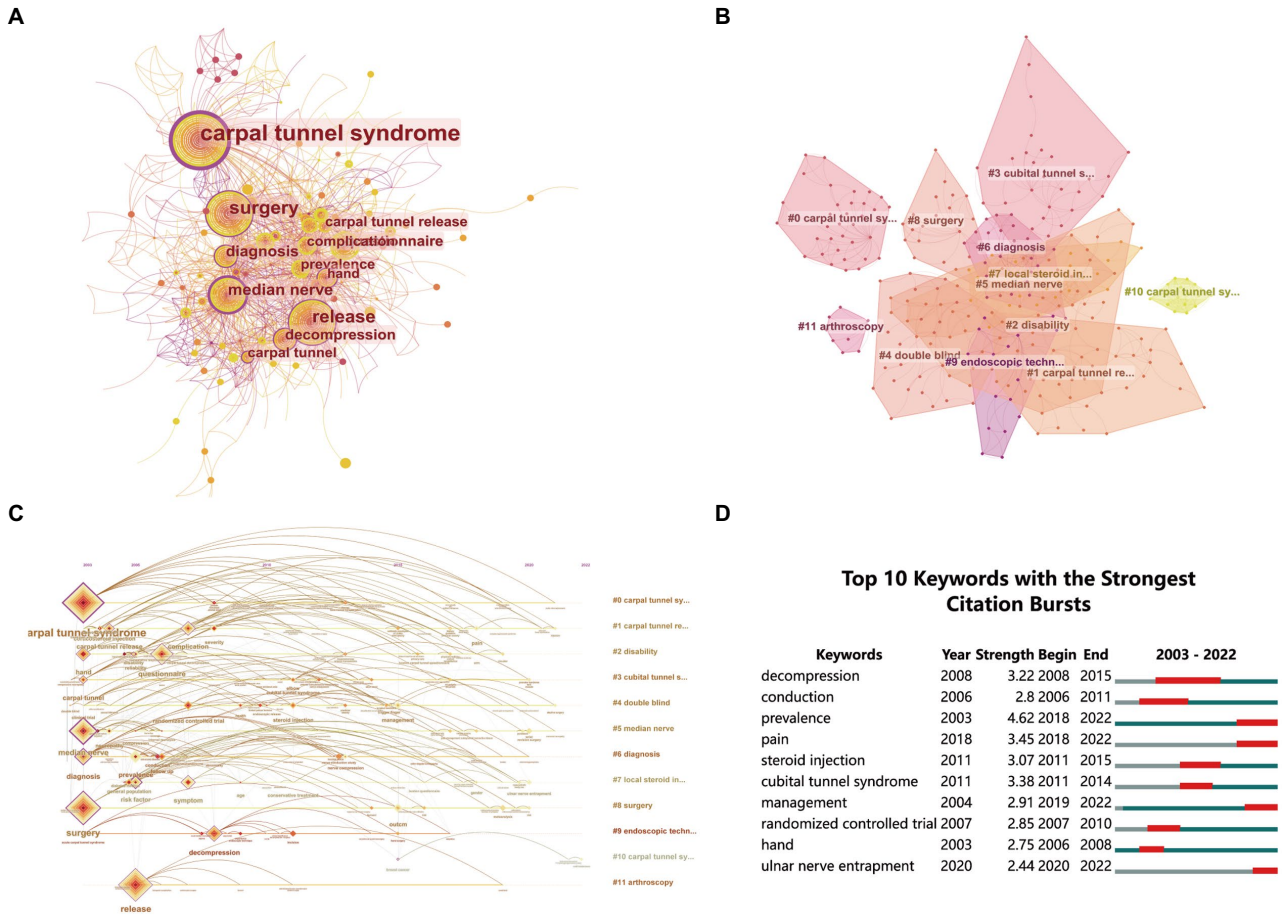
Keyword analysis of research areas on surgical treatment of CTS from 2003 to 2022. **(A)** Keyword co-occurrence network. **(B)** Keyword clustering analysis. **(C)** Keyword clustering timeline diagram. **(D)** The plot of keyword burst curves.

Keyword co-occurrence plots (Figure 4A) were obtained from keyword analysis in the literature using CiteSpace software. Modularity *Q* = 0.7602, with modularity 0.4–0.8 usually forming n small natural network clusters that satisfy the analysis requirements. Weighted mean silhouette *S* = 0.7579, when *S* ≥ 0.5, clustering is generally considered more *S* = 0.7579, when *S* ≥ 0.5, the clustering is generally considered more reasonable and meets the analysis requirements. Each node represents a keyword, the size of the node represents the frequency of the keyword, the linkage of two nodes represents the co-occurrence of two keywords, the color of the linkage represents the time, and the thickness represents the degree. Based on frequency and centrality, we found the following popular keywords: carpal tunnel syndrome, surgery, release, median nerve, diagnosis, questionnaire, decompression, complication, prevalence carpal tunnel release, diagnosis, risk factor, breast cancer, carpal tunnel, complication, decompression, and release (Table 2). It can be seen that the main focus of research on surgical treatment of CTS is on the release of carpal tunnel, changes in median nerve function, prognosis and the mechanism of CTS development.

**Table 2:** TOP-10 of frequency, TOP-10 of centrality.

Rank	Frequency	Label	Rank	Centrality	Label
1	192	Carpal tunnel syndrome	1	0.51	Median nerve
2	76	Surgery	2	0.45	Carpal tunnel syndrome
3	57	Release	3	0.26	Diagnosis
4	36	Median nerve	4	0.23	Surgery
5	28	Diagnosis	5	0.21	Risk factor
6	25	Questionnaire	6	0.2	Breast cancer
7	23	Decompression	7	0.17	Carpal tunnel
8	20	Complication	8	0.16	Complication
9	20	Prevalence	9	0.14	Decompression
10	19	Carpal tunnel release	10	0.13	Release

Keyword co-occurrence network for research areas on surgical treatment of carpal tunnel syndrome from 2003 to 2022.

The keywords were analyzed by clustering (Figure 4B) and the keywords were clustered into 12 LABELS with the clustering labels derived from the LSI/LLR/MI algorithm and the number of clusters was inversely proportional to the size of the clusters. These clustered keywords can reflect the research hotspots in surgical treatment of CTS which can guide the development pattern of this research and new directions of research.

The keyword timeline analysis of the literature was obtained using CiteSpace software to obtain the keyword timeline mapping (Figure 4C). The analysis showed that the pre-research direction mainly focused on research related to the pathogenesis of CTS, with keywords around CTS, surgery, release, median nerve, and diagnosis, which we believe is since after the third technological revolution, especially since 2000, human production and lifestyle have The significant increase in the incidence of soft tissue disorders of the neck and upper limbs (15) following the third technological revolution has led to an increased interest in CTS among surgeons. Since 2005, the field of endoscopic surgery for CTS has evolved, although endoscopic surgery for CTS was reported by Okutsu as early as 1987 (16). A gee and Chow reported on two commonly used endoscopic techniques, the single-channel technique (17) and the dual-channel technique (18). In the last decade, researchers have continued to explore the best treatment modalities for moderate-to-severe CTS, including the use of a single-channel technique and a dual-channel technique. In the last decade, researchers have continued to explore the best treatment modalities for moderate to severe CTS, including drug injection therapy, individualized treatment, and the exploration of risk factors (Table 3).

**Table 3:** Keyword clustering labels for research areas on surgical treatment of carpal tunnel syndrome from 2003 to 2022.

Cluster-ID	Size	Label (LSI)	Label (LLR)	Label (MI)	Average year
#0	34	Carpal tunnel syndrome	Manual therapy (26.91, 1.0E-4)	National trend (2.03)	2013
#1	23	Carpal tunnel release	Longitudinal open incision (19.45, 1.0E-4)	Ulnar nerve (0.09)	2008
#2	21	Carpal tunnel syndrome	Sex difference (21.81, 1.0E-4)	Carpal tunnel syndrome (0.12)	2001
#3	20	Carpal tunnel syndrome	Long-term follow-up (24.07, 1.0E-4)	Parallel-group trial (0.28)	2011
#4	20	Short-term effect	Short-term effect (21.22, 1.0E-4)	Treatment decision (0.4)	2017
#5	20	Median nerve	Micro-invasive us-guided carpal tunnel release (20.91, 1.0E-4)	Carpal tunnel syndrome (0.11)	2016
#6	19	Sonographic follow-up	Sonographic follow-up (40.31, 1.0E-4)	Electrodiagnostic studies (0.16)	2004
#7	18	Carpal tunnel syndrome	Conservative management (24.54, 1.0E-4)	Carpal tunnel syndrome Symptom severity (0.12)	2003
#8	15	Carpal tunnel syndrome	Surgical treatments-a (13.86, 0.001)	Open carpal tunnel release (0.12)	2006
#9	14	Carpal tunnel syndrome	Occupational disease (21.81, 1.0E-4)	Carpal tunnel syndrome (0.11)	2000
#10	12	Double-blind placebo-controlled trial	Double-blind placebo-controlled trial (21.34, 1.0E-4)	Carpal tunnel syndrome (0.11)	2006
#12	6	Evaluation of patients with carpal tunnel syndrome treated by endoscopic technique	Evaluation (9.82, 0.005)	Carpal tunnel syndrome (0.17)	2010

The keyword bursts in the literature were analyzed using CiteSpace software to obtain the 10 keywords with the highest number of citation bursts (Figure 4D). The analysis showed that the keywords decompression (3.22), conduction (2.8), prevalence (4.62), management (2.91), and hand (2.75) had a long duration, and prevalence (4.62), cubital tunnel syndrome (3.38), and decompression (3.22) had high burst intensities indicating that these keywords were research hotspots in the corresponding periods. Prevalence (4.62), pain (3.45), management (2.91), and ulnar nerve entrapment (2.44) continued their prominence in the past 5 years to date, and all have been hotspots of research in recent years.

### 3.4. Reference co-citation analysis

The top ten most frequently co-cited articles in the field of surgical treatment of CTS research were compiled and listed by CiteSpace software to obtain co-citation and highlighting plots of the literature (Figure 3 and Table 4). In a randomized controlled trial by Gerritsen et al. (19), a non-invasive approach to the treatment of CTS only improved patients’ symptoms and did not change outcomes. This provided direction for those who followed to study the treatment of CT. In a study by Hui et al., the efficacy of decompression surgery and steroid injections for CTS was compared in terms of nerve conduction velocity, symptoms (5 dimensions: pain, numbness, sensory abnormalities, weakness/clumsiness, nocturnal arousal) and grip strength, noting that steroid injections only provided short-term relief of symptoms and did not relieve the degeneration of the median nerve from mechanical compression (20).

**Table 4:** Top-10 most frequently cited articles in the field of surgical treatment of CTS research in total.

Rank	Count	Centrality	Author	Year	Reference	Source
	28	0.13	Gerritsen AAM	2002	Splinting vs. surgery in the treatment of carpal tunnel syndrome 10.1245	JAMA-J AM MED ASSOC
2	21	0.26	Hui ACF	2005	A randomized controlled trial of surgery vs. steroid injection for carpal tunnel syndrome	NEUROLOGY
3	21	0.04	Agarwal V	2005	A prospective study of the long-term efficacy of local methylprednisolone acetate injection in the management of mild carpal tunnel syndrome	RHEUMATOLOGY
4	20	0.03	Muller Monique	2004	Effectiveness of hand therapy interventions in primary management of carpal tunnel syndrome: a systematic review	J HAND THER
5	17	0.1	Aroori Somaiah	2008	Carpal tunnel syndrome	ULSTER MED J
6	17	0.05	Baysal O	2006	Comparison of three conservative treatment protocols in carpal tunnel syndrome	INT J CLIN PRACT
7	17	0.04	Atroshi I	2007	The SF-6D health utility index in carpal tunnel syndrome	J HAND SURG-BRIT EUR
8	17	0.02	Greenslade JR	2004	Dash and Boston questionnaire assessment of carpal tunnel syndrome outcome: what is the responsiveness of an outcome questionnaire?	J HAND SURG-BRIT EUR
9	16	0.05	Atroshi I	2013	Methylprednisolone injections for the carpal tunnel syndrome	ANN INTERN MED
10	16	0.02	Bland JDP	2001	Do nerve conduction studies predict the outcome of carpal tunnel decompression?	MUSCLE NERVE

## 4. Discussion

### 4.1. Analysis of global publication trends

A total of 336 articles were obtained from the Web of Science database according to our selection criteria. After importing the 336 articles into the CiteSpace (6.1 R4) software, we performed deduplication and cleaning of the data and found zero duplicates, and a total of 336 articles were extracted for analysis. All articles were published in 179 journals by 316 researchers from 157 countries/regions.

Through bibliometric and visual analysis, we found that the number of publications has increased each year between 2003 and 2022, while more predictive analysis shows that the field is now in a growth phase. Future publication trends are positive.

Research on national/regional cooperation networks can help to promote teamwork and global cooperation in specific areas. In this study, we can see that the country with the highest number of publications is the USA (*n* = 114), followed by the UK (*n* = 24) and Japan (*n* = 21). Furthermore, the USA has the highest centrality at 0.66, indicating that it plays an important role as a bridge in inter-country cooperation. Based on this current situation, strengthening communication and cooperation between national institutions is of profound importance in promoting further development in this field.

The analysis of author collaboration networks and author co-citations helps to analyze the direction of authors’ research and provides further guidance. All 336 articles originated from 316 researchers. The study shows that many scholars have formed collaborative networks centered on themselves and have a more consistent article output. Overall, there is a lack of collaboration between scholars and teams, and enhanced communication and cooperation between authors could have far-reaching implications in promoting further development in the field. Future author collaboration could produce more good quality articles.

An analysis of journals can help researchers choose the right journal to submit their papers. Of the top 10 journals in terms of the number of publications, JCR Q2 and above accounted for five, with the highest IF being COCHRANE DATABASE OF SYSTEMATIC REVIEWS (IF = 12.008). The study shows that surgical treatment of CTS research has greater clinical value and is favored by many high-quality, high-impact journals.

### 4.2. Analysis of the current state of research on surgical treatment of CTS

Surgery is currently the mainstay of the response to moderate to severe CTS. Pressure on the median nerve is relieved by releasing the contents of the carpal tunnel by severing the transverse carpal ligament (21). This gain can be achieved both through open surgery and through small incisions, and later endoscopic release of the carpal tunnel has also been shown to be efficacious. Between 2003 and 2022, researchers have worked to find the most appropriate means of treating CTS. Through nerve conduction velocity, symptoms [the efficacy of different procedures, or surgical versus non-surgical procedures, has been compared in terms of nerve conduction velocity, symptoms (5 dimensions: pain, numbness, sensory abnormalities, weakness/clumsiness, nocturnal arousal)], and grip strength, as well as the most appropriate treatment modalities for different risk factors (20, 22, 23). Thus, the study of the efficacy of different treatment modalities for CTS has been a hot topic in the field.

### 4.3. Prospective analysis of research on surgical treatment of CTS

Current research on surgical treatment of CTS relies mainly on randomized controlled trials, and there is a lack of effective animal models and *in vitro* trials. If future researchers can establish reliable animal models, the mechanism of the development of CTS will be clearer and will better assist surgeons in their clinical decisions. In addition, little information has been reported on the relationship between histological analysis and single-cell sequencing of CTS and surgery, which we believe could better explain the pathogenesis of CTS and provide guidance to clinicians.

### 4.4. Limitations

In this study, we only used CiteSpace software for visual analysis of the Web of Science database; therefore, this strategy may have missed papers published in other databases. Furthermore, the search strategy was created to obtain the most comprehensive data possible, which does not guarantee that all included articles are fully relevant to the study topic. It is known that older literature has a higher chance of being cited and confirmed, which may result in a time lag where recent groundbreaking research has not received sufficient attention. In addition, the software is temporarily unable to capture all authorship information and distinguish the order of authors, which may lead to a less accurate assessment of the contributions of researchers in the field. Therefore, we need to take a dialectical view of the strengths and limitations of this approach compared to traditional reviews.

## Author contributions

DZ conceived the study and carried out the data analysis, interpretation, and manuscript writing. ZW helped to draft the manuscript. LL and SC helped to check the data. JC contributed to methodology and writing—review and editing. All authors contributed to the article and approved the submitted version.

## Conflict of interest

The authors declare that the research was conducted in the absence of any commercial or financial relationships that could be construed as a potential conflict of interest.

## Publisher’s note

All claims expressed in this article are solely those of the authors and do not necessarily represent those of their affiliated organizations, or those of the publisher, the editors and the reviewers. Any product that may be evaluated in this article, or claim that may be made by its manufacturer, is not guaranteed or endorsed by the publisher.
